# Who’s Hurt Most by Economic Shock? Exploring Heterogeneity in the Health-Related Effects of Wealth Loss

**DOI:** 10.1093/geroni/igaa057.1953

**Published:** 2020-12-16

**Authors:** Joseph Wolfe

**Affiliations:** University of Alabama at Birmingham, Birmingham, Alabama, United States

## Abstract

Prior research finds evidence of an effect of negative economic shocks on health, but this growing area has not fully investigated variation in this effect. A large number of people from diverse backgrounds experience a substantial financial setback of some type, and differences related to one’s gender, race/ethnicity, and socioeconomic status (SES) may influence the consequences of economic shocks on one’s life such that the health-related effects of shocks vary systematically in the U.S. population. Thus, this study aims to identify the effects of multiple economic shocks on health in middle adulthood, and whether the effects of shocks on health vary by one’s underlying propensity to experience the shock. The analysis uses newly developed statistical techniques from causal inference literature and over twenty-five years of biographical information from the NLSY-79. Results from the analysis help shed light on important variation in the association between negative economic shocks and health.

